# Special Issue “Bio-Nano Interactions 2.0”

**DOI:** 10.3390/ijms25031667

**Published:** 2024-01-30

**Authors:** Aura Cencini, Massimiliano Magro

**Affiliations:** Department of Comparative Biomedicine and Food Science, University of Padua, Viale dell’Università 16, 35020 Legnaro, Italy; aura.cencini@phd.unipd.it

In 1961, USA’s blues legend Howlin’ Wolf released the single entitled “Down in the Bottom” (Figure 1). Coincidentally, a couple of years before, physicist Richard Feynman expressed the ante litteram concept of nanotechnologies in his famous 1959 lecture at Caltech entitled “There’s Plenty of Room at the Bottom”, in which he stated the following: “I am, as I said, inspired by the biological phenomena in which chemical forces are used in repetitious fashion to produce all kinds of weird effects (one of which is the author). The principles of physics, as far as I can see, do not speak against the possibility of maneuvering things atom by atom”.

Indeed, since the dawn of nanoscience (i.e., the study of nano-sized materials), the aim of nanoscientists has been to emulate the biological system’s degree of complexity, endowing synthetic materials with extraordinary properties, such as the aforementioned ability of “maneuvering things atom by atom”, which is actually what enzymes do since life origin on this planet. The development of nanotechnologies played its part in trying to make Feynman’s vision come true, and it is exciting to realize how far they have gone since their beginning. Nevertheless, more than 60 years later, the burning question is as follows: Are the nanodevices we create today able to manipulate matter at an atomic scale in the fashion that nature does? Nature provides many examples of highly sophisticated nanoengineering by transforming inanimate ingredients, such as oxygen, phosphorus, hydrogen, nitrogen, and so on, into superb life architectures, such as cells, organs, and creatures [1].

Besides the imitation of biological systems, science has been investing a lot of work in understanding how to synergically integrate biology and nanotechnology. In recent years, aiming at biomedical applications, lots of efforts have been dedicated towards joining modern biology and nanotechnology. Both these fields had a profound impact on many other branches of science and technology, with their developments becoming common in our everyday lives, from vaccines and drugs to electronic devices and solar cells. Whilst biology is the study of complex systems that already work and are functional, nanotechnology takes materials of nanometric size and tunes their chemical and physical properties to achieve different functionalities. Since a great part of biological functions takes place at the nanoscale, it is possible to strategically manipulate the activities of biological machineries using nanotechnologies. An increasing number of examples of their use in such a designed and purposeful manner is provided by nanomedicine [2]. From this union, a lot of great things can be made, where the final material is more than just the sum of its parts, giving rise to emerging properties such as chirality at the nanoscale [3]. The interconnections can be multiple: not only is it possible to modify biological (organic) components to change the functioning of a host system, but it is also possible to change inorganic materials and give them a biological identity, creating actual novel biological entities for drug delivery and medical devices for diagnosis and therapy [4]. Despite nanomedicine’s recent advances, there are significant challenges and limitations in the realization of its full potential and its application to a wider range of diseases and medical conditions. Some of these challenges include premature uptake and clearance by the liver and inefficient delivery to target tissues [5]. To address these key challenges, attention has been focused on understanding how nanoengineered materials work in a biological environment. To do this, the study of bio-nano interactions is of the uttermost importance. These can be defined as the interactions between nanoscale materials and biological systems such as biomolecules, but also cells and organisms. Indeed, a complete profile of bio-nano interfaces and interactions would have a great impact on the optimization and de novo design of new nanomaterials, allowing, in turn, for better in vivo performance [6]. Nevertheless, the tuning of nanomaterials’ chemical-physical characteristics also affects how they interact with biological molecules, and also has an impact on their transport, delivery, and function. The parameters of colloidal nanoparticles that are usually considered are size, shape, and surface chemistry, but their stiffness has also been recently investigated for modulating bio-nano interactions [7]. In the same way, the state of cells, tissues, or biological models can also influence the behavior of nanomaterials at the bio-interface as well as their intracellular processing. Until a nanomaterial can interact with its target cells and exert its function, it must go through complex biological matrices and the local physiological environment of the target site [8]. Hence, understanding how the biological factors can be addressed or exploited can guide the design of better and more tailored nanomaterials to improve their functioning as nanomedicines, with the aim of working with and not against biology. Exerting a control at the nanoscale is key, which has already promoted progresses for the development of nanotechnologies able to self-organize for tissue regeneration [9]. In this context, the use of engineered biomolecules, such as nucleic acids, enabled us to develop self-assembled hybrid nano-objects with machine-like properties, using simple Watson–Crick base-pairing rules [10,11]. On the other hand, the synthesis of inorganic nanomaterials with pseudo-enzymatic properties (nanozymes) allowed us to establish novel devices able to manipulate matter at the nanoscale level, leading nanoscientists a step forward with regard to mimicking nature [12,13]. Furthermore, besides synthetic strategies, analytical innovation provided us with the possibility to observe materials approaching the atomic resolution, e.g., through scanning probe microscopy (SPM) [1]. The convergence of all these novelties had already resulted in a revolution in classic medicine. Hence, it is clear that the objective of nanoscientists is to build things the way nature does: atom by atom and molecule by molecule. The truth is that the “bottom” dreamed by Feynman still remains a largely unexplored landscape of challenges and opportunities. Taking inspiration from the “repetitious fashion” of biological entities, nanomaterials can be ideally synthesized atom by atom and can provide periodicities and repetitive motifs for tailoring novel pseudo-biological features. As Howlin’ Wolf sung “meet me in the bottom, bring me my running shoes”, this goal can be reached only step by step.

## Figures and Tables

**Figure 1:**
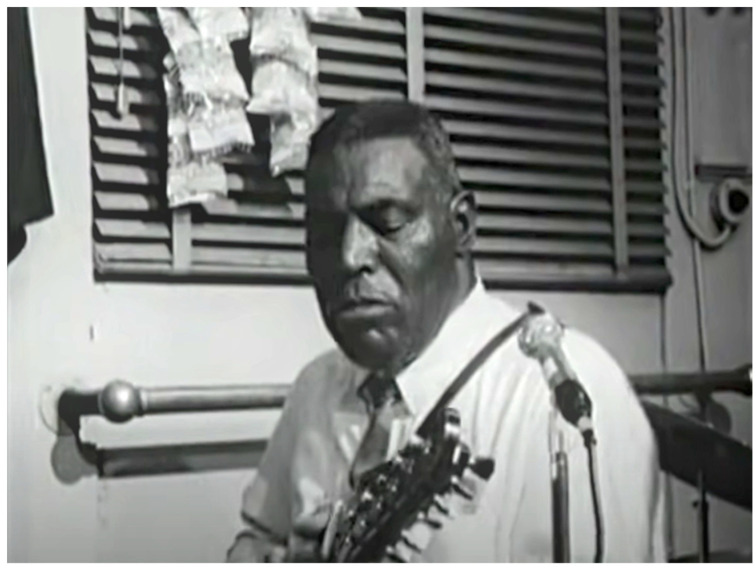
The great Howlin’ Wolf performing “Down in the Bottom” live.
